# Class, type and position of 9148 surgically removed third molars in 3206 patients: A retrospective study

**DOI:** 10.4317/medoral.17548

**Published:** 2011-12-06

**Authors:** Benjamín Morales-Trejo, Miriam L. Rocha-Navarro, Anselmo L. Acosta-Veloz, Angélica Juárez-Hernández

**Affiliations:** 1 Degree in Dental Surgery. Specialist in Oral and Maxillofacial Surgery. Coordinator of the Department of Oral and Maxillofacial Surgery. Department of Oral and Maxillofacial Surgery, Dental School, Salle Bajío University, A.C. León, Guanajuato. Mexico; 2 Degree in Dental Surgery. Master and Doctorate in Medical Sciences. Professor – Investigator. Department of Oral and Maxillofacial Surgery, Dental School, Salle Bajío University, A.C. León, Guanajuato. Mexico; 3 Degree in Dental Surgery. Department of Oral and Maxillofacial Surgery, Dental School, Salle Bajío University, A.C. León, Guanajuato. Mexico

## Abstract

Objective: To investigate the class, type, position, diagnosis and most common procedures used in the surgical removal of third molars, and evaluate the sex and age distribution in a representative sample of Mexican patients. Study Design: A retrospective descriptive study was made covering the period 1993-2008 in relation to 9148 extracted third molars in 3206 patients treated in the Dental School of Salle Bajío University, A.C. (Mexico). Patients of either sex and aged 11-59 years, with at least one third molar programmed for surgical removal, were included in the study. A descriptive statistical study was made. Results: The mean patient age was 27.6 ± 10.6 years. There were 2093 females (65.3%) and 1111 males (34.6%). In relation to the 4025 upper molars, extraction was decided for prophylactic reasons in 3827 cases (95.08%). Type A presentations were recorded in 1929 cases (47.9%), with a vertical position in 1931 teeth (48%). In relation to the 5123 lower third molars, extraction was likewise most often indicated for prophylactic reasons (4424 cases, 86.36%). A total of 2353 teeth corresponded to type A (45.9%), 2545 were class I cases (49.7%), and a mesioangular position was observed in 1850 cases (36.1%). Conclusions: The present study shows that in Mexican patients, upper third molars most often correspond to type A and class I, with a vertical position, while lower third molars predominantly correspond to type A and class I, with a mesioangular position. This information can help dental surgeons take better decisions before and after surgery, to the benefit of their patients.

** Key words:**Third molars, retrospective review, surgical removal.

## Introduction

Surgical removal of third molars (TMs) is one of the most common dentoalveolar procedures in oral and maxillofacial surgery (1,2). Extraction is decided due to the presence of pathological situations such as infection, non-restorable caries, cysts, tumors and the destruction of adjacent teeth and bone (3).

In view of the great demand for treatments of this kind, it is very important for the dental surgeon to know the most common class, type and position pattern associated to TM extractions in the population, since such information conditions the presurgical preparation of the patient, the choice of surgical technique and the identification of risk factors which thus can be foreseen preoperatively – contributing to reduce and/or avoid complications during and after the operation, and affording faster and more satisfactory patient recovery (4).

This is the first study Mexican study to describe the most frequent class, type and position of upper and lower TMs programmed for surgical removal. The study sample moreover is considered to be representative of the Mexican general population.

## Material and Methods

A retrospective descriptive study involving data collection from case histories was made, covering the period 1993-2008, in relation to patients treated in the Department of Oral and Maxillofacial Surgery of the Dental School of Salle Bajío University, A.C. (Mexico). Patients of either sex and aged 11-59 years, with at least one third molar programmed for surgical removal, were included in the study. The subjects were systematically classified as follows: type I (healthy individuals or patients with disease antecedents posing no risk for dental treatment) or type II (patients with controlled disease antecedents or individuals in which risk for dental treatment could be avoided). Totally edentulous individuals requiring only the removal of a TM were excluded from the study.

The study was evaluated and approved by the local Ethics Committee. Patient data confidentiality was guaranteed.

All patients included in the study were subjected to panoramic X-ray study. We recorded the depth of the TMs with respect to the occlusal plane (type A, B, C), along with the distance between the ascending ramus of the mandible or superior tuberosity of the upper maxilla and the distal surface of the second molar (class I, II, III) according to the classification of Pell and Gregory, described by Almendros-Marqués et al. (5). In addition, we recorded molar angulation with respect to the longitudinal axis of the second molar (mesioangular, distoangular, vertical, horizontal), based on the classification of Winter, as described by Almendros-Marqués et al. (6). However, in some cases involving doubt as to the type, class or position of the molars, classification was carried out based on tracings and measurements on the X-rays.

All surgical extractions of the TMs were carried out by three maxillofacial surgeons, who determined the classification of the molars from the panoramic X-ray data. The surgeons were calibrated on the basis of measurements in triplicate of molars randomly selected in patients not included in the study. Calibration proved acceptable when the results were seen to be identical in over 85% of the cases. All extractions were made under local anesthesia (with premedication in some cases) using a conventional surgical technique. Upper TMs were accessed with the raising of an oral flap and, where necessary, bone was removed with a low-speed handpiece under continuous irrigation. Flap suturing was not carried out, except in those cases presenting communication with the maxillary sinus. In the case of the lower TMs we raised a vestibular flap with releasing incisions to the second molar. Where necessary, bone was removed and the crown and roots were sectioned. In all these cases the flap was repositioned with 4/0 silk suture. The patients were instructed on postoperative care, including the use of ice, diet, oral hygiene, general care and medications use. The sutures were removed after 7 days.

The data were processed using the Stat Soft statistical package (Tulsa, AZ, USA). A descriptive analysis was made to determine the central tendency and dispersion measures of the variables. Frequency tables were used to determine patient age and sex distribution, as well as the type, class and position of the extracted TMs.

TM extraction was indicated for prophylactic reasons when the patient presented one or more of the following findings: malpositioning, lack of space, periodontal involvement of the second molar, and caries affecting the second molar.

## Results

A total of 9148 TMs were removed from 3206 patients, of which 1111 were males and 2093 females (34.65% and 65.28% respectively). The mean age at the time of extraction was 27.66 ± 10.62 years. The most prevalent age group corresponded to 18-25 years, with 1469 patients (16.05%) and 3709 extracted TMs (40.54%). In turn, 53.71% of the patients (n = 1722) were of type I, while 36.09% (n = 1157) corresponded to type II. Most of the TMs (6087 teeth, 66.54%) were removed adopting a surgical or trans-alveolar approach, while 3059 teeth (31.5%) were removed using an intra-alveolar approach. There were 4025 upper molar extractions (44%) and 5123 lower molar extractions (56%). The most frequently removed tooth was number 48 (2572 extractions, 28.11%), followed by 38 (2551 extractions, 27.88%), 28 (2026 extractions, 22.14%), and number 18 (1999 extractions, 21.85%).

The most frequent reason for extraction was prevention (8251 cases, 90.19%), followed by chronic pericoronitis (532 cases, 5.82%), acute pericoronitis (336 cases, 3.67%) and orthodontic indications (29 cases, 0.32%). The molars with the most frequent diagnoses of acute and chronic pericoronitis were the lower TMs, with 279 (5.45%) and 404 cases (7.9%), respectively, versus 57 (1.42%) and 128 cases (3.18%) among the upper molars. The tooth with the most frequent diagnosis of acute pericoronaritis was number 38 (147 cases, 5.45%), while number 48 was the tooth most often diagnosed with chronic pericoronitis (204 cases, 7.89%).

Class I was the most frequent presentation for both the upper and lower TMs. Specifically, class I was identified in 2600 cases (55.66%), followed by class II in 1702 cases (36.44%), and class III in 369 cases (7.90%).

As regards the depth of the TMs with respect to the occlusal plane, 4282 teeth corresponded to type A (54.17%), 2320 to type B (29.34%), and 1303 to type C (16.48%), for both the upper and lower TMs ( Table 1).

**Table 1:**
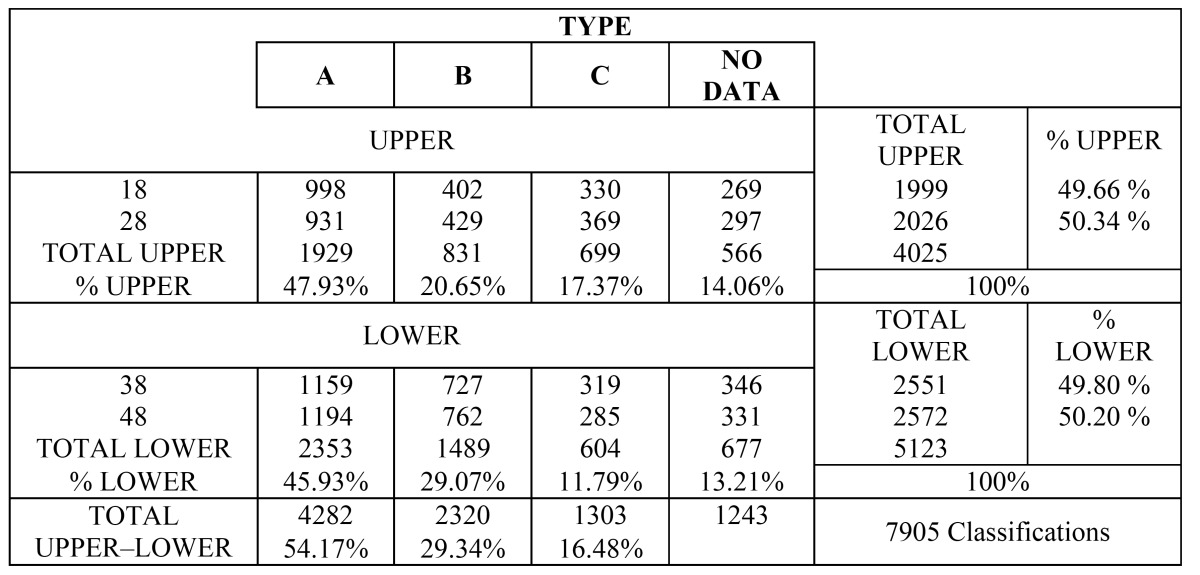
Frequency of upper and lower third molar types according to the classification of Pell and Gregory.

In relation to TM position, differences were observed between the upper and lower molars. In effect, in the upper TMs the most frequent position was vertical (1931 cases, 48.0%), while in the lower TMs the most common position was mesioangular (1850 cases, 36.1%). In addition, there were 11 ectopic presentations (0.12%) - 6 corresponding to upper TMs (0.065%) and 5 to lower TMs (0.054%) ( Table 2).

**Table 2:**
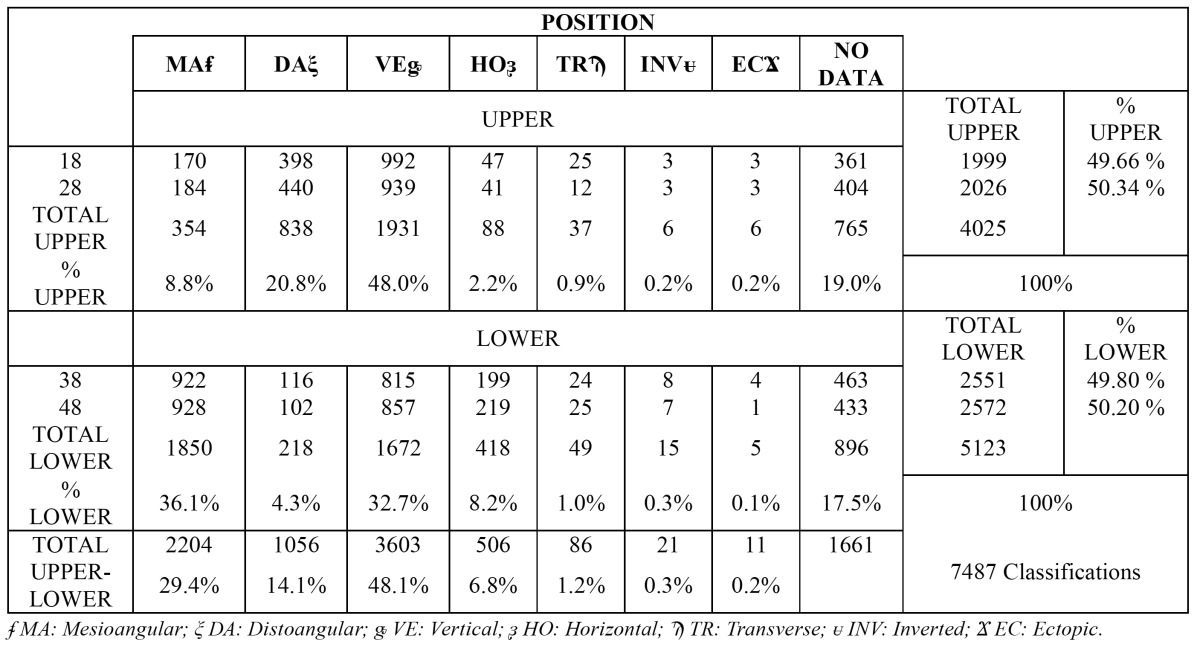
Frequency of upper and lower third molar positions according to the classification of Winter.

## Discussion

The international literature describes third molar (TM) extraction as the most common procedure in maxillofacial surgery. Due to the many indications and the important demand for TM removal, emphasis should be placed on the need for continued training and improvement of the academic and technical processes for ensuring correct extraction (1,2,4).

In the present study, the mean age of the Mexican patients subjected to TM extraction was similar to that reported by Grossi et al. (7) in 213 Italian patients requiring 266 extractions. In Canada, Blondeau et al. (8) recorded an age range of 12-55 years, with an average of 24.4 years. Based on the above, on comparing with the Italian and Canadian studies, our own data coincide in terms of the average age at which individuals require TM removal.

Regarding gender distribution, there are a number of indicators as to why females were more prevalent in our study: (a) a proportionately larger number of females are seen in the dental clinic; (b) women are more aware and concerned about their dental care; and (c) males tend to auto-medicate themselves and thus postpone or delay dental treatment. Blondeau et al. (8), in a series of 327 patients, recorded a 58.4% prevalence of females, in coincidence with our own observations. Chaparro-Avendaño et al. (9) also reported a similar percentage, since a full 66.9% of their 390 surgical extractions corresponded to females. In contrast, however, Bataineh et al. (10) reported a 57.7% prevalence of males (out of 1282 patient), probably because these individuals had greater access to dental care than the females.

In the present study, the majority of extractions were decided for prophylactic reasons, followed by chronic periodontitis, acute periodontitis and orthodontic indications. This coincides with the data obtained by Linden et al. (11), though in contrast Chaparro-Avendaño et al. (9) reported more frequent indication for orthodontic reasons, followed by prophylaxis. This suggests that Mexican patients are more prone to seek help for functional problems such as dental malpositioning, lack of space or periodontal disease in the area of the second molar, than for solving aesthetic problems.

We also found that there is sufficient space between the ascending ramus of the mandible and the distal surface of the second molar to house the entire mesiodistal diameter of the crown of the third molar, since class I was the most frequent presentation for both the upper and lower TMs, in coincidence with the observations of Figueiredo et al. (12) in the Spanish population.

Regarding the position of the upper TM, we recorded a greater prevalence of vertical presentations, in coincidence with the findings published by Chaparro-Avendaño et al. (9), though Kruger et al. (13) found the mesioangular to be the most frequent presentation, followed by the vertical position. We therefore can deduce that the Mexican and Spanish populations are similar in this sense.

In the case of the lower TM, we recorded a greater prevalence of mesioangular presentations, followed by the vertical position, in coincidence with the data reported by Chaparro-Avendaño et al. (9), Figueiredo et al. (12), Kruger et al. (13) and Poeschl et al. (14). Similar observations apply to the United States, where Gbotolorun et al. (15) conducted a three-year prospective study in which the mesioangular position was seen to be the most frequent presentation in 331 extracted lower TMs corresponding to 329 patients. In this same population, Haug et al. (16) compiled the data of 63 maxillofacial surgeons who performed a total of 8333 TM extractions in 3760 patients, and likewise found the mesioangular position to be the most common presentation. However, Bataineh et al. (10) found the vertical and horizontal positions to be the most common presentations. This suggests that the different conditions relating to patient and gender selection could explain the differences observed between studies.

In conclusion, third molar extraction is more frequent in females than in males, and the mean patient age at the time of extraction largely coincides with the data found in the literature. Our study shows that third molar extraction is most often indicated for prophylactic reasons, followed by infection. The Mexican population presents characteristics in relation to third molar class, type and position similar to those found on other continents. The clinical and radiological criteria for establishing the most common class, type and position in Mexican patients allow the dental surgeon in general practice to plan treatment on the basis of an adequate assessment of the difficulty of extraction, and to weigh the option of referring the patient to a specialist if necessary.
